# Rare *MLL*-*ELL* fusion transcripts in childhood acute myeloid leukemia—association with young age and myeloid sarcomas?

**DOI:** 10.1186/s40164-016-0037-2

**Published:** 2016-03-05

**Authors:** Ioannis Panagopoulos, Ludmila Gorunova, Gitte Kerndrup, Signe Spetalen, Anne Tierens, Liv T. N. Osnes, Kristin Andersen, Lil-Sofie Ording Müller, Marit Hellebostad, Bernward Zeller, Sverre Heim

**Affiliations:** 1 Section for Cancer Cytogenetics, Institute for Cancer Genetics and Informatics, The Norwegian Radium Hospital, Oslo University Hospital, Nydalen, P.O.Box 4953, 0424 Oslo, Norway; 2 Centre for Cancer Biomedicine, Faculty of Medicine, University of Oslo, Oslo, Norway; 3 Department of Pathology, Aarhus University Hospital, Aarhus, Denmark; 4 Department of Pathology, Oslo University Hospital, Oslo, Norway; 5 Laboratory Medicine Program, Department of Haematopathology, University Health Network, Toronto, Canada; 6 Department of Immunology, Oslo University Hospital, Oslo, Norway; 7 Department of Radiology, Oslo University Hospital, Oslo, Norway; 8 Department of Pediatrics, Drammen Hospital, Vestre Viken HF, Drammen, Norway; 9 Department of Pediatrics, Oslo University Hospital, Oslo, Norway; 10 Faculty of Medicine, University of Oslo, Oslo, Norway

**Keywords:** Acute myeloid leukemia, Myeloid sarcoma, RNA-sequencing, Chromosomal translocation, t(11;19)(q23;p13), *MLL*, *ELL*, Fusion gene

## Abstract

**Background:**

The chromosomal translocation t(11;19)(q23;p13) with a breakpoint within subband 19p13.1 is found mainly in acute myeloid leukemia (AML) and results in the *MLL*-*ELL* fusion gene. Variations in the structure of *MLL*-*ELL* seem to influence the leukemogenic potency of the fusion in vivo and may lie behind differences in clinical features. The number of cases reported so far is very limited and the addition of more information about *MLL*-*ELL* variants is essential if the possible clinical significance of rare fusions is to be determined.

**Case presentation:**

Cytogenetic and molecular genetic analyses were done on the bone marrow cells of a 20-month-old boy with an unusual form of myelomonocytic AML with multiple myeloid sarcomas infiltrating bone and soft tissues. The G-banding analysis together with FISH yielded the karyotype 47,XY, +6,t(8;19;11)(q24;p13;q23). FISH analysis also demonstrated that *MLL* was split. RNA-sequencing showed that the translocation had generated an *MLL*-*ELL* chimera in which exon 9 of *MLL* (nt 4241 in sequence with accession number NM_005933.3) was fused to exon 6 of *ELL* (nt 817 in sequence with accession number NM_006532.3). RT-PCR together with Sanger sequencing verified the presence of the above-mentioned fusion transcript.

**Conclusions:**

Based on our findings and information on a few previously reported patients, we speculate that young age, myelomonoblastic AML, and the presence of extramedullary disease may be typical of children with rare *MLL*-*ELL* fusion transcripts.

**Electronic supplementary material:**

The online version of this article (doi:10.1186/s40164-016-0037-2) contains supplementary material, which is available to authorized users.

## Background

The chromosomal translocation t(11;19)(q23;p13) has been reported in both acute myeloid (AML) and acute lymphoblastic leukemia (ALL) [1]. Breakpoints within subband 19p13.3 are found in both ALL (primarily in infants and children) and AML with the translocation t(11;19)(q23;p13.3) leading to the fusion of *MLL* with *MLLT1* (also known as *ENL, LTG19*, and *YEATS1*) generating an *MLL*-*MLLT1* fusion gene [2]. Breakpoints within subband 19p13.1 are found mostly in AML where the translocation t(11;19)(q23;p13.1) results in the *MLL*-*ELL* fusion gene [3]. *MLL*-*ELL* fusions were recently found also in two biphenotypic leukemias [4]. Two other *MLL*-fusion genes have also been reported in t(11;19)-positive AML. A recurrent *MLL*-*MYO1F* [translocation t(11;19)(q23;p13.2)] fusion gene was seen in infant AML [5, 6], whereas an *MLL*-*SH3GL1* fusion [translocation t(11;19)(q23;p13.3)] was reported in a case of childhood AML [7].

In the majority of *MLL*-*ELL* fusion transcripts, exon 9, 10, 11 or 12 of *MLL* is fused to exon 2 of *ELL* [3, 8–13]. A variant form of *MLL*-*ELL* fusion transcript has been reported in chronic myelomonocytic leukemia in which *MLL* exon 9 (exon 10 according to Nilson et al. [14]) was fused to *ELL* exon 3 [15]. Furthermore, in a case of congenital acute monoblastic leukemia with a three-way translocation t(1;19;11)(p36;p13.1;q23), De Braekeler et al. showed that the genomic breakpoints in *MLL* and *ELL* occurred in introns 9 and 5, respectively [8, 16].

The leukemogenic potency of *MLL*-*ELL* fusion genes was demonstrated in murine model systems [17]. Moreover, variant forms of *MLL*-*ELL* were shown to impair transforming activities in vitro [12]. These observations suggest that variations in *MLL*-*ELL* structure may influence leukemogenic potency of the fusion also in vivo, and they hint that such variability may be behind variation in clinical features. Because so few such cases have been reported, the addition of more cases with *MLL*-*ELL* variants is essential if the possible clinical significance of rarer fusions is to be determined. In the present study, we report a childhood leukemia in which a three-way translocation caused the fusion of exon 9 of *MLL* with exon 6 of *ELL*. To the best of our knowledge, this is only the second case in which exon 6 of *ELL* was found to be fused to *MLL* [8, 16, 18].

## Case presentation

### Ethics statement

The study was approved by the regional ethics committee (Regional komité for medisinsk forskningsetikk Sør-Øst, Norge, http://helseforskning.etikkom.no), and written informed consent was obtained from the patient’s parents to publication of the case details. The ethics committee’s approval included a review of the consent procedure. All patient information has been anonymized.

### Clinical presentation

**Fig. 1:**
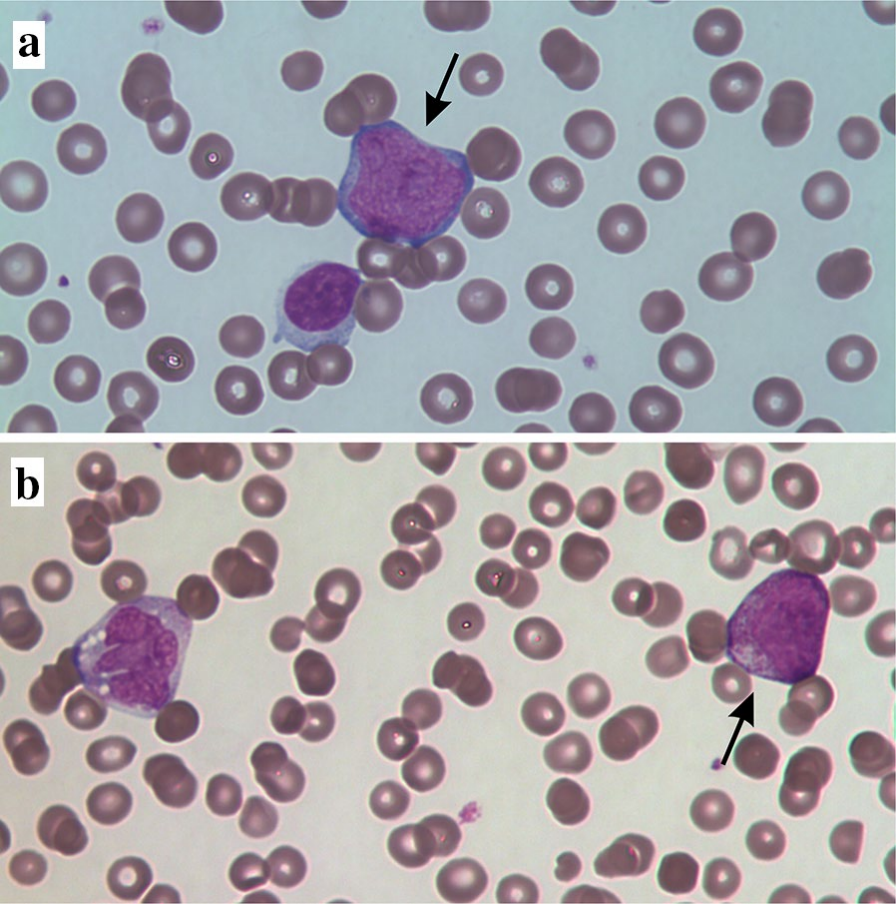
Microscopic picture of peripheral blood smear of the AML patient from the time of admission to the local pediatric department showing **a** a myeloblast (*arrow*) and a lymphocyte. **b** A promyelocyte (*arrow*) and a vacuolated monocyte. Magnification 100×

A 20-month-old, previously healthy boy presented with intermittent pain in the hip. The boy’s general condition was reduced, he was pale and weak, and passive movements in the right hip were painful. His spleen was slightly enlarged, whereas liver size was normal. Blood tests revealed anemia (hemoglobin 8.2 g/dL), thrombocytopenia (64 × 10^9^/L), normal white blood cell count (11.9 × 10^9^/L) with monocytosis (1.5 × 10^9^/L), and immature myeloid cells were seen in the blood smear (Fig. 1).

**Fig. 2:**
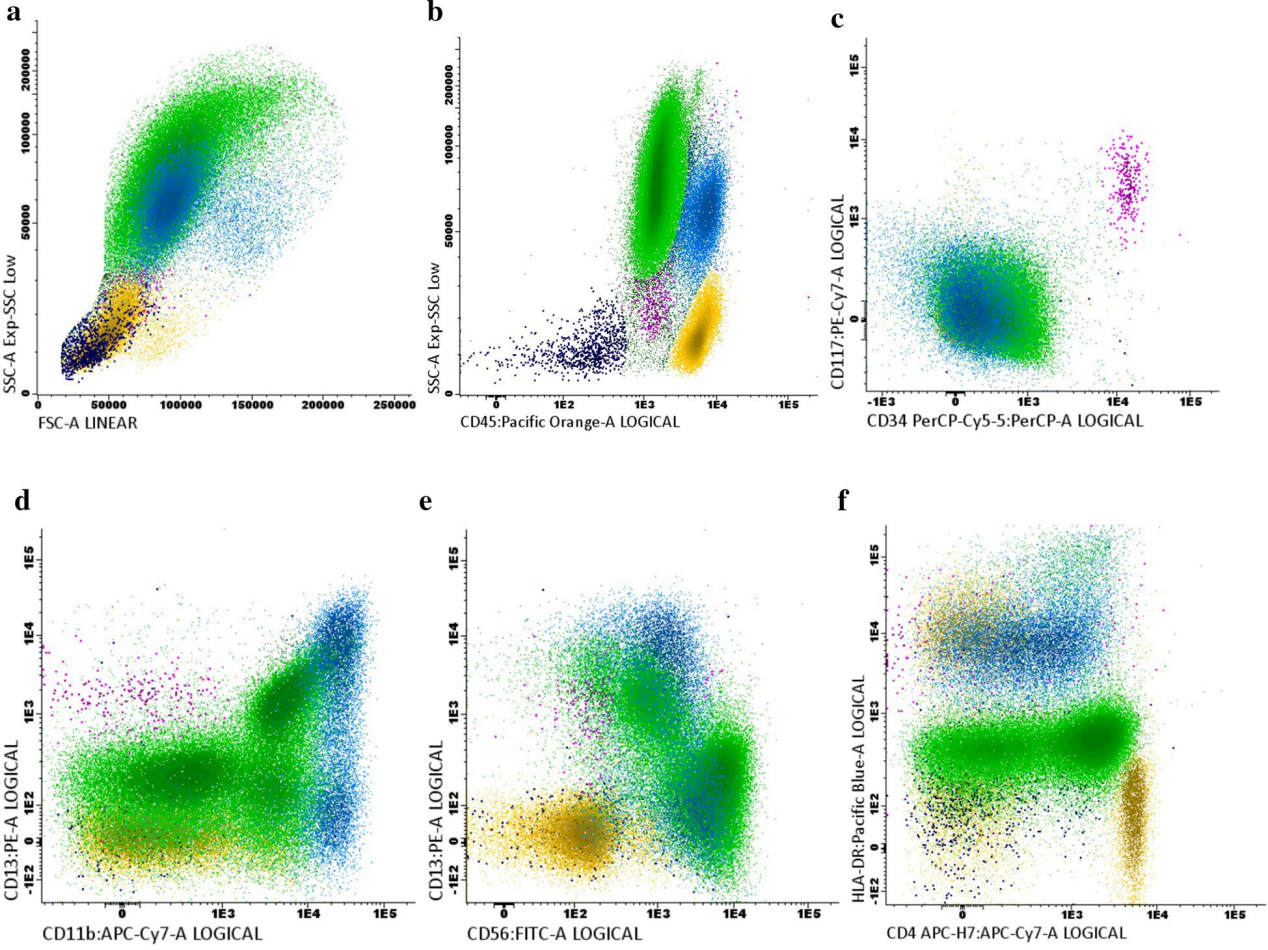
Bivariate *dot plots* of the flow cytometric analysis of the bone marrow aspirate. The* color codes* for the different cell subsets are as follows: *green* for granulocytes, *blue* for monocytes, *purple* for CD34 positive precursors, *yellow* for lymphocytes, and *dark blue* for erythroid precursors.* Dot plot* **a** and **b** show normal light scatter signals and normal expression of CD45 of all cell populations. No increase of CD34/CD117 positive cells was demonstrated as can be seen in* dot plot* **c**.* Dot plot* **d** shows partial loss of CD13 expression on monocytes as well as on granulocytes and shift to immaturity of the granulocytes of which the majority were negative for CD11b. The aberrant expression of CD56 by granulocytes and monocytes is demonstrated in* dot plot* **e**. Immature granulocytes were positive for CD4 as shown in* dot plot* **f**

A bone marrow investigation (which was repeated several times due to diagnostic difficulties) and a biopsy from one of the bony lesions revealed morphologically a myeloid hyperplasia comprised of predominantly monomorphonuclear cells. Erythropoiesis and megakaryopoiesis were sparse without dysplasia and decreased further with time until they were nearly absent in the later biopsies. The monomorphonuclear cells were positive for CD4 and CD56. The proportion of CD34+ and CD117+ cells was not increased. Further, flow cytometry analysis (Fig. 2) confirmed the normal distribution of CD34^+^ or CD117^+^ precursor cells but revealed two distinct myeloid populations comprised of 68 % CD15 ^bright +^/HLA-DR^−^ granulocytic cells and 15 % CD64 ^bright +^/HLA-DR^−^ monocytic cells. Both lineages showed marker expression consistent with a shift to early stages as well as abnormal expression of CD56. In addition, CD13, a common myeloid marker, was not expressed in the early stages of these myeloid populations whereas abnormal expression of CD4 was demonstrated on early granulocytes. Only 50 % of the granulocytes had acquired expression of CD11b and to a lesser extent CD16 which is consistent with maturation beyond the promyelocyte stage. However, CD10^+^/CD16^+^ end-stage neutrophils were not identified. Around 20 % of the monocytes were negative for CD35 and CD14 which are features of promonocytes. Based on the above immunophenotypic findings alone, a definitive diagnosis of acute myeloid leukemia could not be made.

**Fig. 3:**
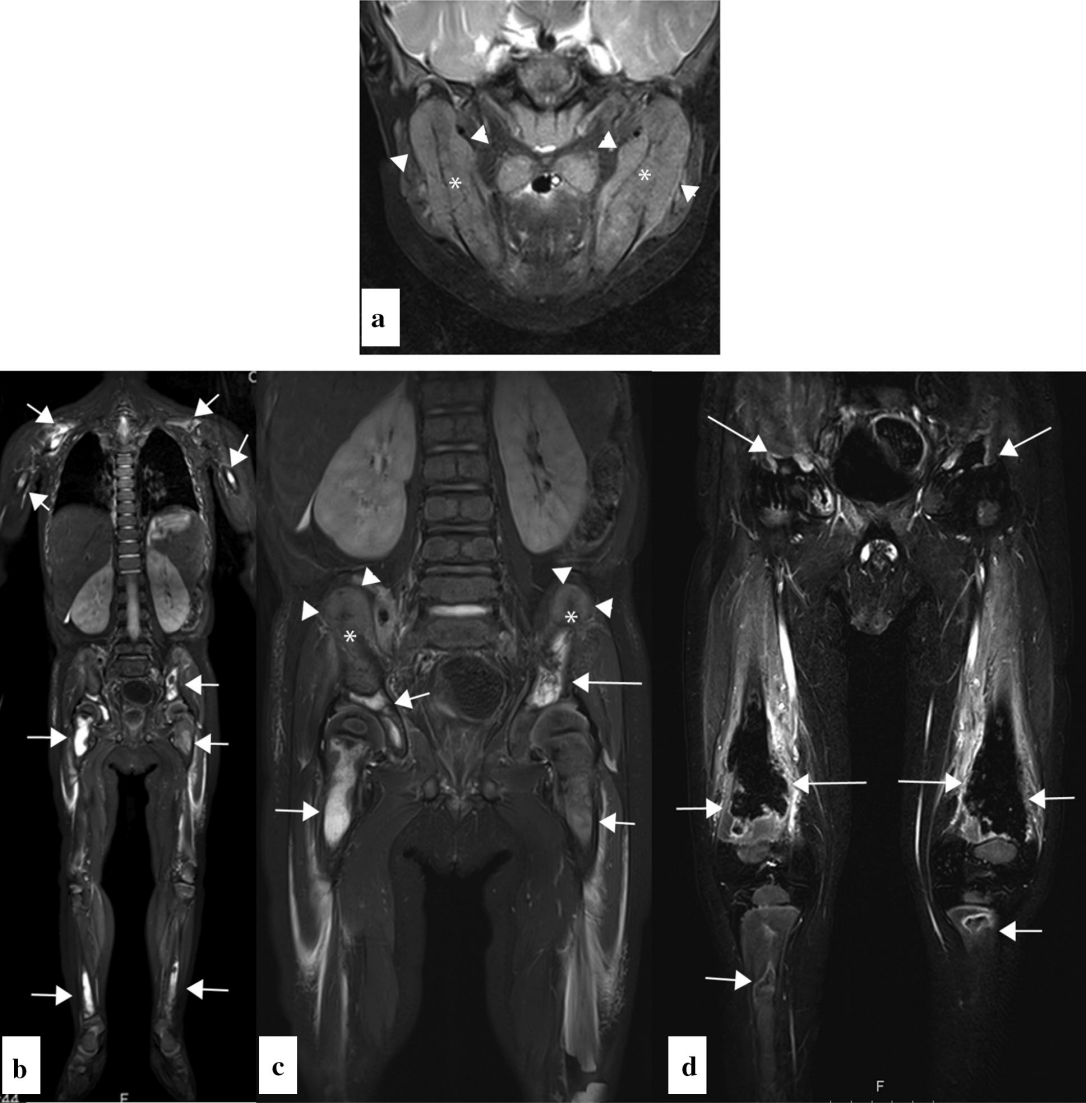
Coronal water-sensitive, fat-suppressed MRI-sequences of the mandible (**a**), whole-body (**b**), pelvis, and femurs of the AML patient (**c**, **d**) showing extensive infarcts in several bones (*arrows*). In the mandible and iliac wings, there was marked, spiculated cortical reaction and new bone formation (*arrowheads*) with destruction of the underlying skeleton (*asterisk*). Subtracted post contrast T1 fat suppressed images of the pelvis and femurs showed low-signal avascular areas with a peripheral rim of enhancement caused by the multiple infarcts (*arrows*). There was soft tissue edema in the lower extremities. The kidneys were slightly enlarged

Whole body magnetic resonance imaging (WBMRI) showed marked spiculated periosteal reaction and new bone formation in the mandible, iliac bones, and bilaterally in the distal femur (Fig. 3a–d). In addition, there were widespread bone lesions with infarctions and necroses involving the skull base, mandible, scapulae, pelvic bones, femurs, and tibia. There was soft tissue involvement of the lower extremities with edema in the muscles and subcutaneous tissue and fluid along the facial planes (Fig. 3a–d).

**Fig. 4:**
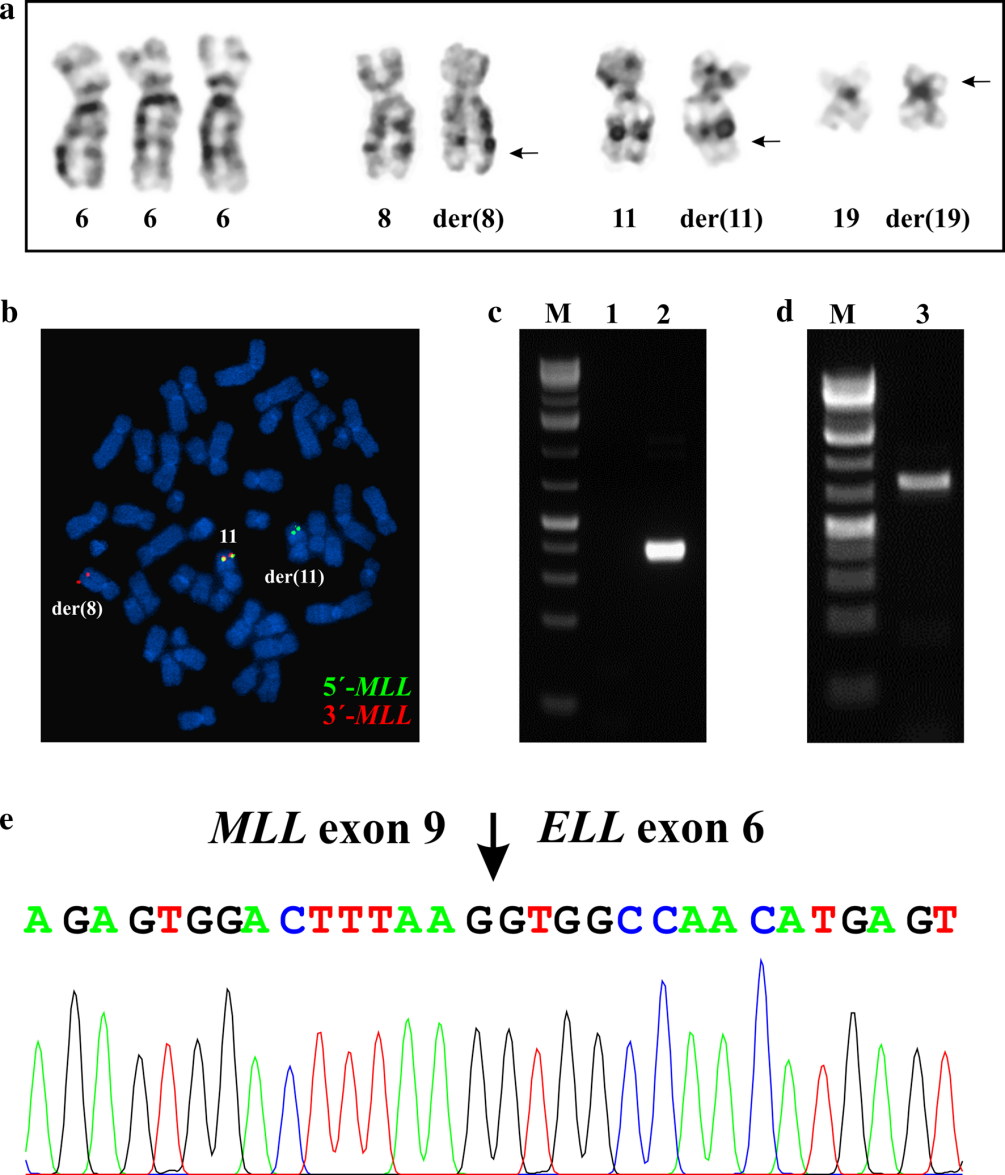
Cytogenetic, FISH, and PCR analyses of the AML patient. **a** G-banded karyotype showing trisomy 6, der(8), der(11), and der(19) of the t(8;19;11)(q24;p13;q23) together with the corresponding normal chromosome homologs; breakpoint positions are indicated by *arrows*. **b** FISH using an *MLL* breakapart probe showed rearrangement of *MLL*. The 3´-end part of the MLL gene (*red probe*) has moved to the q arm of the der(8), while the 5´-end part of the gene (*green probe*) remains on 11q23 of the der(11). **c** The initial RT-PCR amplifications for the detection of a possible *MLL*-*ELL* fusion transcript. *Lane 1*, nested PCR with the forward primers located in exon 7 of *MLL* and reverse primers located in exon 4 of *ELL* (MLL-3947F1/ELL-415R) failed to amplify any cDNA fragments. *Lane 2*, amplification of a cDNA fragment of the *ABL1* gene using the primers ABL1-91F1 and AsBL1-404R1 suggested that the synthesized cDNA was of good quality. **d** RT-PCR using a new reverse primer located in exon 8 of *ELL* (primer ELL-1044R1) and a forward primer located in exon 7 of MLL (primer MLL-3878F) amplified a cDNA fragment. M, 1 Kb DNA ladder. **e** Partial sequence chromatogram of the amplified fragment using the primers MLL-3878F and ELL-1044R1 showing the junction of the *MLL*-*ELL* chimeric transcript

The G-banding analysis of bone marrow metaphase cells at diagnosis showed 11 cells carrying an extra chromosome 6 as well as the chromosomal translocation t(11;19)(q23;p13) yielding the karyotype 47,XY, +6,t(11;19)(q23;p13) [11] (Fig. 4a).

Interphase FISH analyses of bone marrow cells using the Cytocell multiprobe ALL panel (Cytocell, http://www.cytocell.co.uk/) showed a split signal of the *MLL* locus in 146 out of 201 investigated interphase nuclei (data not shown). FISH analysis of metaphase spreads using the *MLL* breakpoint probe (Cytocell, http://www.cytocell.co.uk/) showed that the red signal (distal) had moved not to chromosome 19 but to the q arm of chromosome 8 (Fig. 4b). Thus, the modified karyotype after G-banding analysis and FISH was 47,XY, +6,t(8;19;11)(q24;p13;q23) [11] (Fig. 4a, b).

Mainly on the basis of the detected *MLL*-rearrangement, we interpreted the boy’s disease as an unusual form of myelomonocytic AML with multiple myeloid sarcomas infiltrating bone and soft tissues.

During the investigation, the patient’s extensive skeletal lesions increased causing therapy-resistant pain and his general condition deteriorated. After 3 weeks, AML-directed therapy was begun according to the NOPHO-DBH AML 2012 protocol [19]. He received five courses (MEC, ADxE, HAM, HA3E, FLA) at 5–6 weeks intervals. For details, see Additional file 1: Figure: S1.

Clinically, the boy recovered rather quickly. Evaluation before course two showed no remaining *MLL*-rearranged cells by FISH in the bone marrow. MRI controls of his bony lesions demonstrated continuous, but slow, regression. At 9 months after cessation of treatment, the boy is clinically healthy. As expected, MRI still shows several small residual bone lesions undergoing regression. Unexpectedly, however, the patient has developed a new, small (about 5 %) clone in the bone marrow with a solitary 7q deletion. This may or may not represent an emerging secondary malignancy [20–23], and the situation is being monitored closely to see whether the clone expands and gives rise to hematologically recognizable disease.

### Initial RT-PCR experiments

Total RNA, isolated from the patient’s bone marrow at the time of diagnosis, was reverse-transcribed using iScript Advanced cDNA Synthesis Kit for RT-qPCR (Bio-Rad Laboratories, Oslo, Norway) and cDNA corresponding to 20 ng total RNA was used as template in PCR amplifications as previously described [24, 25]. The initial RT-PCR with the primer set MLL-3878F/ELL-498R1 as well as nested PCR with the primers MLL-3947F1/ELL-415R (the sequences of the primers are listed in Table 1) failed to amplify any cDNA fragments (Fig. 4c). At the same time, use of the primer set ABL1-91F/ABL1-404R1 led to amplification of an *ABL1* cDNA fragment suggesting that the synthesized cDNA was of good quality (Fig. 4c).

**Table 1:** Primers used for PCR amplification and Sanger sequencing analyses

Name	Sequence (5´– >3´)	Direction	Position/exon	Reference sequence	Gene
MLL-3735F	CCCATCAGCAAGAGAGGATCCTGC	Forward	3758-3781/7	NM_005933.3	*MLL (KMT2A)*
MLL-3878F	AGTCAAGCAAGCAGGTCTCCCAGC	Forward	3901-3924/7	NM_005933.3	*MLL (KMT2A)*
MLL-3947F1	GCCACCTACTACAGGACCGCCAAG	Forward	3947-3970/7	NM_005933.3	*MLL (KMT2A)*
ELL-415R1	GGCACACACCGTGATCTTGTCCTG	Reverse	438-415/4	NM_006532.3	*ELL*
ELL-498R1	TTGATGACAATGGCACTTCGGCTC	Reverse	498-521/4	NM_006532.3	*ELL*
ELL-960R1	TCCAAGGAGGCTGCCAGTGCTC	Reverse	981-960/7	NM_006532.3	*ELL*
ELL-1044R1	CGATGAAATCAGGAGGCTGCAGC	Reverse	1066-1044/8	NM_006532.3	*ELL*
ABL1-91F1	CAGCGGCCAGTAGCATCTGACTTTG	Forward	280-304/2	NM_005157.5	*ABL1*
ABL1-404R1	CTCAGCAGATACTCAGCGGCATTGC	Reverse	617-593/3	NM_005157.5	*ABL1*

### RNA-sequencing

Because of the negative RT-PCR results, the less than typical cytogenetic findings, and the clinical picture, three µg of the total RNA extracted from the patients’ bone marrow at the time of diagnosis were subjected to high-throughput paired-end RNA-sequencing at the Norwegian Sequencing Centre, Oslo University Hospital (http://www.sequencing.uio.no/) as described elsewhere [24, 26]. The raw sequencing data were subsequently analyzed using FusionCatcher which is a program designed to detect fusion genes from high throughput sequencing data [27]. More than 100 potential fusion transcripts were found (Additional file 2: Table: S1), among them an *MLL*-*ELL* in which exon 9 of *MLL* (nt 4241 in sequence with accession number NM_005933.3) was fused to exon 6 of *ELL* (nt 817 in sequence with accession number NM_006532.3). No reciprocal *ELL*-*MLL* fusion transcript was found.

In order to verify the fusion obtained with FusionCatcher, we used the “grep” command utility [28] to search for expressions composed of 10 nt of *MLL* and 10 nt of *ELL* upstream and downstream of the fusion point, respectively (Table 2). Using the expression “GACTTTAAGGTGGCCAACAT” which is composed of 10 nt, “GACTTTAAGG”, from *MLL* and 10 nt, “TGGCCAACAT”, from *ELL*, 38 sequences were retrieved (Table 2).

**Table 2:** Sequences retrieved with the «grep» command using the expression “GACTTTAAGGTGGCCAACAT”

ATGGAGTCCACAGGATCAGAGTGGACTTTAAGGTGGCCAACATGAGTGCTAAGGACGGCACGTGTACACTGCAGGACTGCATGTACAAGGATGTGCAGAAGGACTGGCCTGGCTACTCGGAGGGGG
GTTCTAAGCAAAAAATTCCAGCAGATGGAGTCCACAGGATCAGAGTGGACTTTAAGGTGGCCAACATGAGTGCTAAGGACGGCACGTGTACACTGCAGGACTGCATGTACAAGGATGTGCAGAAGG
AAAAAATTCCAGCAGATGGAGTCCACAGGATCAGAGTGGACTTTAAGG**TGGCCAACATGAGTGCTAAGGACGGCACGTGTACACTGCAGGACTGCATGTACAAGGATGTGCAGAAGGACTGGCCTG**
CCTCAGCACTCTCTCCAATGGCAATAGTTCTAAGCAAAAAATTCCAGCAGATGGAGTCCACAGGATCAGAGTGGACTTTAAGG**TGGCCAACATGAGTGCTAAGGACGGCACGTGTACACTGCAGGA**
GCACTCTCTCCAATGGCAATAGTTCTAAGCAAAAAATTCCAGCAGATGGAGTCCACAGGATCAGAGTGGACTTTAAGG**TGGCCAACATGAGTGCTAAGGACGGCACGTGTACACTGCAGGACTGCA**
AATGCAGGCACTTTGAACATCCTCAGCACTCTCTCCAATGGCAATAGTTCTAAGCAAAAAATTCCAGCAGATGGAGTCCACAGGATCAGAGTGGACTTTAAGG**TGGCCAACATGAGTGCTAAGGAC**
GTGGACTTTAAGG**TGGCCAACATGAGTGCTAAGGACGGCACGTGTACACTGCAGGACTGCATGTACAAGGATGTGCAGAAGGACTGGCCTGGCTACTCGGAGGGGGACCAGCAGCTGCTGAAGCGG**
CCTCAGCACTCTCTCCAATGGCAATAGTTCTAAGCAAAAAATTCCAGCAGATGGAGTCCACAGGATCAGAGTGGACTTTAAGG**TGGCCAACATGAGTGCTAAGGACGGCACGTGTACACTGCAGGA**
CCTCAGCACTCTCTCCAATGGCAATAGTTCTAAGCAAAAAATTCCAGCAGATGGAGTCCACAGGATCAGAGTGGACTTTAAGG**TGGCCAACATGAGTGCTAAGGACGGCACGTGTACACTGCAGGA**
CTCTCTCCAATGGCAATAGTTCTAAGCAAAAAATTCCAGCAGATGGAGTCCACAGGATCAGAGTGGACTTTAAGG**TGGCCAACATGAGTGCTAAGGACGGCACGTGTACACTGCAGGACTGCATGT**
CTCTCTCCAATGGCAATAGTTCTAAGCAAAAAATTCCAGCAGATGGAGTCCACAGGATCAGAGTGGACTTTAAGG**TGGCCAACATGAGTGCTAAGGACGGCACGTGTACACTGCAGGACTGCATGT**
GAACATCCTCAGCACTCTCTCCAATGGCAATAGTTCTAAGCAAAAAATTCCAGCAGATGGAGTCCACAGGATCAGAGTGGACTTTAAGG**TGGCCAACATGAGTGCTAAGGACGGCAAGTGTACACT**
TGAACATCCTCAGCACTCTCTCCAATGGCAATAGTTCTAAGCAAAAAATTCCAGCAGATGGAGTCCACAGGATCAGAGTGGACTTTAAGG**TGGCCAACATGAGTGCTAAGGACGGCACGTGTACAC**
TCTCCAATGGCAATAGTTCTAAGCAAAAAATTCCAGCAGATGGAGTCCACAGGATCAGAGTGGACTTTAAGG**TGGCCAACATGAGTGCTAAGGACGGCACGTGTACACTGCAGGACTGCATGTACA**
TGGACTTTAAGG**TGGCCAACATGAGTGCTAAGGACGGCACGTGTACACTGCAGGACTGCATGTACAAGGATGTGCAGAAGGACTGGCCTGGCTACTCGGAGGGGGACCAGCAGCAGATCGGAAGAG**
TGGACTTTAAGG**TGGCCAACATGAGTGCTAAGGACGGCACGTGTACACTGCAGGACTGCATGTACAAGGATGTGCAGAAGGACTGGCCTGGCTACTCGGAGGGGGACCAGCAGCAGATCGGAAGAG**
CTTTGAACATCCTCAGCACTCTCTCCAATGGCAATAGTTCTAAGCAAAAAATTCCAGCAGATGGAGTCCACAGGATCAGAGTGGACTTTAAGG**TGGCCAACATGAGTGCTAAGGACGGCACGTGTA**
CAGGATCAGAGTGGACTTTAAGG**TGGCCAACATGAGTGCTAAGGACGGCACGTGTACACTGCAGGACTGCATGTACAAGGATGTGCAGAAGGACTGGCCTGGCTACTCGGAGGGGGACCAGCAGCT**
ATCAGAGTGGACTTTAAGG**TGGCCAACATGAGTGCTAAGGACGGCACGTGTACACTGCAGGACTGCATGTACAAGGATGTGCAGAAGGACTGGCCTGGCTACTCGGAGGGGGACCAGCAGCTGCTG**
GGAGTCCACAGGATCAGAGTGGACTTTAAGG**TGGCCAACATGAGTGCTAAGGACGGCACGTGTACACTGCAGGACTGCATGTACAAGGATGTGCAGAAGGACTGGCCTGGCTACTCGGAGGGGGAC**
GCACTCTCTCCAATGGCAATAGTTCTAAGCAAAAAATTCCAGCAGATGGAGTCCACAGGATCAGAGTGGACTTTAAGG**TGGCCAACATGAGTGCTAAGGACGGCACGTGTACACTGCAGGACTGCA**
GCAATAGTTCTAAGCAAAAAATTCCAGCAGATGGAGTCCACAGGATCAGAGTGGACTTTAAGG**TGGCCAACATGAGTGCTAAGGACGGCACGTGTACACTGCAGGACTGCATGTACAAGGATGTGC**
GTGGACTTTAAGG**TGGCCAACATGAGTGCTAAGGACGGCACGTGTACACTGCAGGACTGCATGTACAAGGATGTGCAGAAGGACTGGCCTGGCTACTCGGAGGGGGACCAGCAGCTGCTGAAGCGG**
ATGCAGGCACTTTGAACATCCTCAGCACTCTCTCCAATGGCAATAGTTCTAAGCAAAAAATTCCAGCAGATGGAGTCCACAGGATCAGAGTGGACTTTAAGG**TGGCCAACATGAGTGCTAAGGACG**
CACAGGATCAGAGTGGACTTTAAGG**TGGCCAACATGAGTGCTAAGGACGGCACGTGTACACTGCAGGACTGCATGTACAAGGATGTGCAGAAGGACTGGCCTGGCTACTCGGAGGGGGACAGATCG**
CTCCAATGGCAATAGTTCTAAGCAAAAAATTCCAGCAGATGGAGTCCACAGGATCAGAGTGGACTTTAAGG**TGGCCAACATGAGTGCTAAGGACGGCACGTGTACACTGCAGGACTGCATGTACAA**
CTCCAATGGCAATAGTTCTAAGCAAAAAATTCCAGCAGATGGAGTCCACAGGATCAGAGTGGACTTTAAGG**TGGCCAACATGAGTGCTAAGGACGGCACGTGTACACTGCAGGACTGCATGTACAA**
CAGGCACTTTGAACATCCTCAGCACTCTCTCCAATGGCAATAGTTCTAAGCAAAAAATTCCAGCAGATGGAGTCCACAGGATCAGAGTGGACTTTAAGG**TGGCCAACATGAGTGCTAAGGACGGCA**
CAGCACTCTCTCCAATGGCAATAGTTCTAAGCAAAAAATTCCAGCAGATGGAGTCCACAGGATCAGAGTGGACTTTAAGG**TGGCCAACATGAGTGCTAAGGACGGCACGTGTACACTGCAGGACTG**
ATCAGAGTGGACTTTAAGG**TGGCCAACATGAGTGCTAAGGACGGCACGTGTACACTGCAGGACTGCATGTACAAGGATGTGCAGAAGGACTGGCCTGGCTACTCGGAGGGGGACCAGCAGCTGCTG**
GTTCTAAGCAAAAAATTCCAGCAGATGGAGTCCACAGGATCAGAGTGGACTTTAAGG**TGGCCAACATGAGTGCTAAGGACGGCACGTGTACACTGCAGGACTGCATGTACAGATCGGAAGAGCGTC**
AATAGTTCTAAGCAAAAAATTCCAGCAGATGGAGTCCACAGGATCAGAGTGGACTTTAAGG**TGGCCAACATGAGTGCTAAGGACGGCACGTGTACACTGCAGGACTGCATGTACAAGGATGTGCAG**
GTTCTAAGCAAAAAATTCCAGCAGATGGAGTCCACAGGATCAGAGTGGACTTTAAGG**TGGCCAACATGAGTGCTAAGGACGGCACGTGTACACTGCAGGACTGCATGTACAAGGATGTGCAGAAGG**
AATAGTTCTAAGCAAAAAATTCCAGCAGATGGAGTCCACAGGATCAGAGTGGACTTTAAGG**TGGCCAACATGAGTGCTAAGGACGGCACGTGTACACTGCAGGACTGCATGTACAAGGATGTGCAG**
CACAGGATCAGAGTGGACTTTAAGG**TGGCCAACATGAGTGCTAAGGACGGCACGTGTACACTGCAGGACTGCATGTACAAGGATGTGCAGAAGGACTGGCCTGGCTACTCGGAGGGGGACCAGCAG**
CAGCACTCTCTCCAATGGCAATAGTTCTAAGCAAAAAATTCCAGCAGATGGAGTCCACAGGATCAGAGTGGACTTTAAGG**TGGCCAACATGAGTGCTAAGGACGGCACGTGTACACTGCAGGACTG**
ATGGAGTCCACAGGATCAGAGTGGACTTTAAGG**TGGCCAACATGAGTGCTAAGGACGGCACGTGTACACTGCAGGACTGCATGTACAAGGATGTGCAGAAGGACTGGCCTGGCTACTCGGAGGGGG**
CTCAGCACTCTCTCCAATGGCAATAGTTCTAAGCAAAAAATTCCAGCAGATGGAGTCCACAGGATCAGAGTGGACTTTAAGG**TGGCCAACATGAGTGCTAAGGACGGCACGTGTACACTGCAGGAC**

The sequences of *ELL* are in bold

### Molecular genetic confirmation of the fusion

PCR with the MLL-3878F and ELL-1044R1 primer combination (Table 1) amplified a fragment from the patient’s bone marrow cDNA (Fig. 4d). Sanger sequencing of the amplified product showed that it was a chimeric *MLL*-*ELL* cDNA fragment in which exon 9 of *MLL* was fused to exon 6 of *ELL*, i.e., the same *MLL*-*ELL* fusion transcript found by RNA-sequencing (Fig. 4e; Table 2).

## Conclusions

We report a case of AML genetically characterized by a three-way translocation, t(8;19;11)(q24;p13;q23), leading to rearrangement of the *MLL* gene and the generation of a chimeric *MLL*-*ELL* transcript with fusion of *MLL* exon 9 to *ELL* exon 6. The initial RT-PCR amplifications relied on forward primers located in exon 7 of *MLL* and reverse primers located in exon 4 of *ELL*; this choice was based on findings in previous studies in which *MLL* was shown to fuse with exon 2 or 3 of *ELL* [3, 10, 11, 15, 29, 30]. The PCRs with these primer sets (first PCR with MLL-3878F/ELL-498R1, then nested PCR with the primers MLL-3947F1/ELL-415R) failed to amplify any cDNA fragments. It was a combination of three methods—banding cytogenetics, FISH, and RNA-sequencing—that helped us identify the present *MLL* exon 9-*ELL* exon 6 fusion. G-banding analysis showed what appeared to be a regular chromosomal translocation t(11;19)(q23;p13) (Fig. 4a), a well-known change in acute leukemia. FISH showed that although *MLL* was split, the distal part of the gene was moved not to the derivative 19 but, surprisingly, to the long arm of chromosome 8 (Fig. 4b). Finally, RNA-sequencing showed that exon 9 of *MLL* was fused to exon 6 of *ELL* (Table 2). RT-PCR using a new reverse primer located in exon 8 of *ELL* (primer ELL-1044R1, MLL-3878F and ELL-1044R1 primer combination) then confirmed the fusion transcript (Fig. 4d, e).

In 2009, De Braekeleer and coworkers reported a case of congenital acute monoblastic leukemia with a three-way translocation, t(1;19;11)(p36;p13.11;q23), which involved the *MLL* gene and generated an *MLL*-*ELL* fusion identical to that of the present case [8, 16, 18]. To the best of our knowledge, these are the only two cases hitherto reported in which exon 6 of *ELL* is fused to *MLL*. It is certainly intriguing that three-way translocations, an unusual phenomenon behind *MLL*-rearrangements, had occurred in both cases; it may hint at some currently hidden mechanism behind the generation of the genomic change. The number of AML cases with *MLL* exon 9-*ELL* exon 6 fusions might actually be underestimated when assessed by means of RT-PCR amplifications using primer sets based on hitherto published studies [3, 10, 11, 15, 29, 30]. This situation may be remedied by use of a new RT-PCR method that includes primers to detect fusion of *MLL* also with exon 6 of *ELL* [18].

**Fig. 5:**
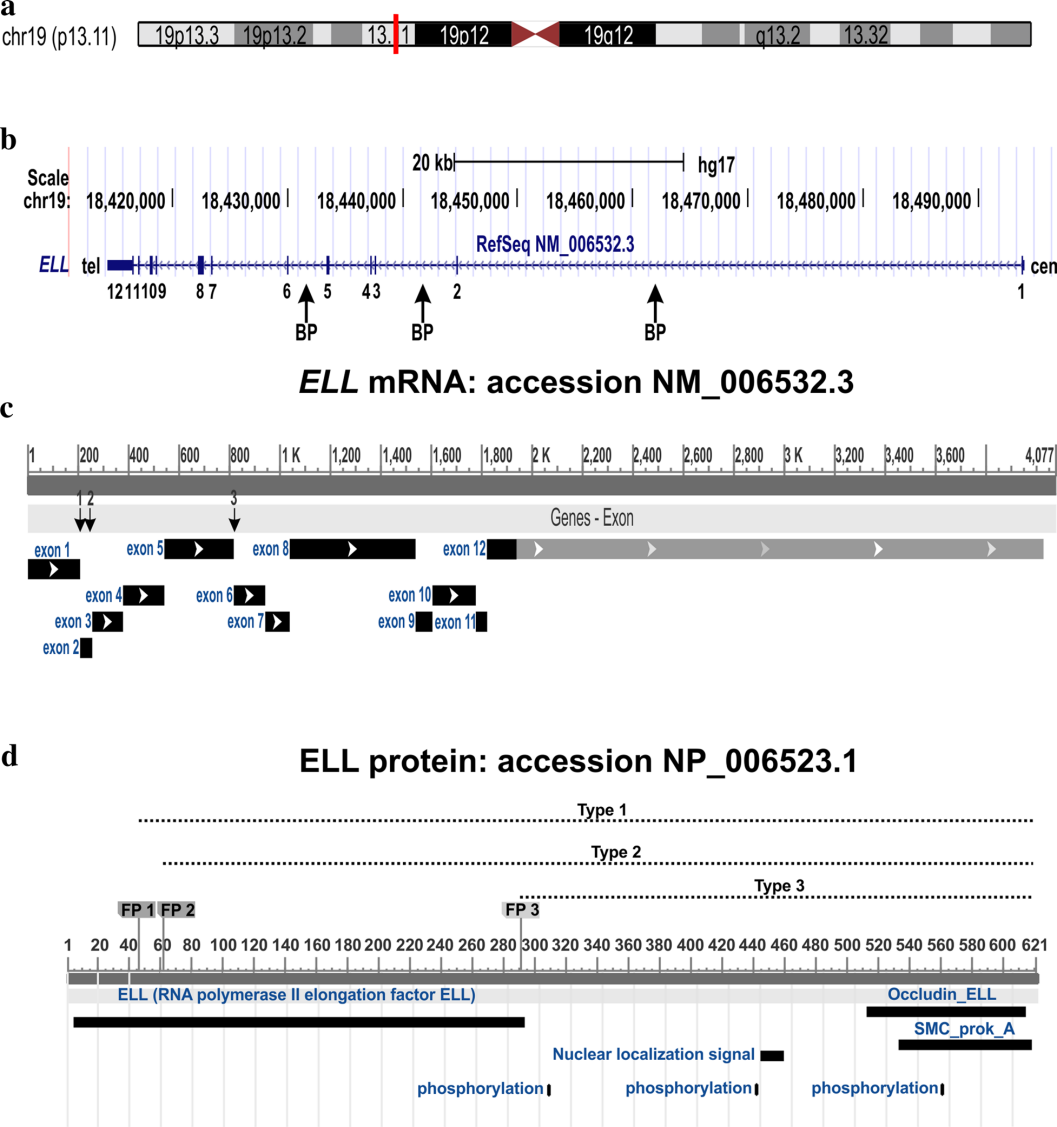
Properties of the *ELL* gene. **a** Ideogram of chromosome 19 showing the location of the *ELL* gene in chromosome subband 19p13.11 (*vertical red line*). **b** Exon intron structure of the *ELL* gene. *Vertical boxes* indicate exons. The breakpoints (*BP*) have been found in introns 1, 2, and 5. *Vertical arrows* indicate the genomic BP regions. The transcription is from centromere (cen) to telomere (tel). **c** Diagram showing mRNA of ELL with accession number NM_006532.3. The exons which code for protein are in *black*. The *vertical arrows* indicate the fusion points 1, 2, and 3 in the *MLL*-*ELL* fusion transcripts. **d** Diagram showing the ELL protein, the known domains, and the phosphorylation sites. FP1, FP2, and FP3 are the fusion points with the MLL in the three types (1, 2, and 3) of MLL-ELL fusion protein. All three MLL-ELL proteins retain the occludin homology domain, the SMC_prok_A domain, the nuclear localization signal, and the three phosphorylation sites

Current knowledge holds that there are three types of *MLL*-*ELL* fusion transcripts (Fig. 5). Type 1, found in the majority of cases, is characterized by *ELL* exon 2 being fused to 5´-*MLL* [3, 8–13]. Type 2 was so far found in only a single case of chronic myelomonocytic leukemia (CMML) that transformed to AML; it had exon 3 of *ELL* fused to 5´-*MLL* [15]. Type 3, of which the present case is one of two, is caused by a three-way translocation leading to the fusion of exon 6 of *ELL* with *MLL* (present case; [8, 16, 18]). All three MLL-ELL fusion proteins contain the amino-terminal region of MLL which includes the AT hooks, the methyltransferase domain, and the repression domain [31].

The case we describe presented unusual clinical features. Most conspicuous were the widespread, very painful bone lesions with soft tissue involvement which were interpreted as multiple myeloid sarcomas and extensive bone infarcts, not mere marrow infiltration. The validity of this diagnosis is supported by the gradual, protracted resolution of these lesions taking place during and after therapy. To the best of our knowledge, only four cases, including the present one, have been reported of very young AML patients displaying myelomonocytic features, myeloid sarcomas, and involvement of the *MLL*-*ELL* fusion gene [8, 16, 32, 33]. The four patients have similar cytogenetic and genetic (*MLL*-*ELL* fusion) features. Three of them had a three-way translocation generating the *MLL*-*ELL* fusion: the present case with t(8;19;11)(q24;p13;q23), a female newborn with t(1;19;11)(p36;p13.11;q23) [16, 32], and a three-month-old boy with t(6;19;11)(p22;p13;q23) [33]. None of the patients had the type 1 *MLL*-*ELL* fusion (see above). In the patient with t(1;19;11)(p36;p13.11;q23), the data suggest an *MLL* exon 9-*ELL* exon 6 fusion transcript, similar to our case [16, 32]. In a two-month-old child reported by De Braekeler et al, the genomic breakpoints in *MLL* and *ELL* indicated an *MLL* exon 9-*ELL* exon 3 fusion transcript [8, 32]. In the three-month-old boy with t(6;19;11)(p22;p13;q23), the translocation resulted in an *MLL* exon 8-*ELL* exon 3 fusion transcript [33].

Due to the small number of patients described, it is impossible to make definite statements about prognosis. Nevertheless, treatment results so far on patients carrying rare *MLL*-*ELL* fusion genes seem to have been encouraging. The newborn patient did not receive antileukemic therapy and died 24 h after birth [32], but the three treated patients, including the present case, went into remission [32, 33]. Two of them seem to be long-term survivors [32, 33], and our patient is in complete clinical remission 1 year after diagnosis. The clinical importance of the small clone with a 7q deletion that has emerged in remission is unclear. Recurrent cytogenetic abnormalities are sometimes seen in AML and ALL patients who are in complete clinical remission and may persist for years in the bone marrow even in the absence of progression to leukemia [20–23]. A wait-and-see approach is therefore prudent.

## Additional files

**Additional file 1: Figure S1.** Treatment overview NOPHO-DBH AML2012 protocol (standard arm).

**Additional file 2: Table S1.** Fusion transcripts detected using FusionCatcher.
