# Supplementary figures and images for: Rare MLL-ELL fusion transcripts in childhood acute myeloid leukemia—association with young age and myeloid sarcomas?
Source: Exp Hematol Oncol. 2016 Mar 5;5:8. doi: 10.1186/s40164-016-0037-2 (PMC4779576; doi:10.1186/s40164-016-0037-2)

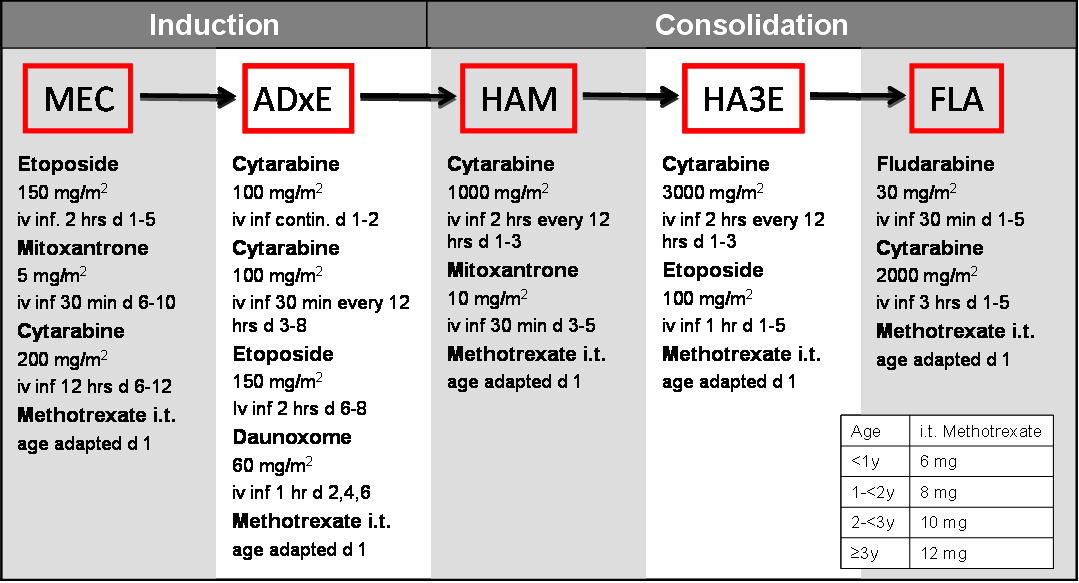

Supplement: Supplementary file 1 — Additional file 1: Figure S1. Treatment overview NOPHO-DBH AML2012 protocol (standard arm). [file 40164_2016_37_MOESM1_ESM.tif]
